# A Bayesian neural network predicts the dissolution of compact planetary systems

**DOI:** 10.1073/pnas.2026053118

**Published:** 2021-10-01

**Authors:** Miles Cranmer, Daniel Tamayo, Hanno Rein, Peter Battaglia, Samuel Hadden, Philip J. Armitage, Shirley Ho, David N. Spergel

**Affiliations:** ^a^Department of Astrophysical Sciences, Princeton University, Princeton, NJ 08 544;; ^b^Department of Physical and Environmental Sciences, University of Toronto at Scarborough, Toronto, ON M1C 1A4, Canada;; ^c^David A. Dunlap Department of Astronomy and Astrophysics, University of Toronto, Toronto, ON M5S 3H4, Canada;; ^d^DeepMind, London EC4A 3TW, United Kingdom;; ^e^Center for Astrophysics, Harvard & Smithsonian, Cambridge, MA 02138;; ^f^Department of Physics and Astronomy, Stony Brook University, Stony Brook, NY 11790;; ^g^Center for Computational Astrophysics, Flatiron Institute, New York, NY 10010;; ^h^Department of Physics, Carnegie Mellon University, Pittsburgh, PA 15217

**Keywords:** deep learning, planetary dynamics, Bayesian analysis, chaos

## Abstract

We introduce a Bayesian neural network model that can accurately predict not only if, but also when a compact planetary system with three or more planets will go unstable. Our model, trained directly from short N-body time series of raw orbital elements, is more than two orders of magnitude more accurate at predicting instability times than analytical estimators, while also reducing the bias of existing machine learning algorithms by nearly a factor of three. Despite being trained on compact resonant and near-resonant three-planet configurations, the model demonstrates robust generalization to both nonresonant and higher multiplicity configurations, in the latter case outperforming models fit to that specific set of integrations. The model computes instability estimates up to $10^{5}$ times faster than a numerical integrator, and unlike previous efforts provides confidence intervals on its predictions. Our inference model is publicly available in the SPOCK (https://github.com/dtamayo/spock) package, with training code open sourced (https://github.com/MilesCranmer/bnn_chaos_model).

## Significance

Despite over 300 y of effort, no solutions exist for predicting when a general planetary configuration will become unstable. We introduce a deep learning architecture to push forward this problem for compact systems. While current machine learning algorithms in this area rely on scientist-derived instability metrics, our new technique learns its own metrics from scratch, enabled by a internal structure inspired from dynamics theory. Our model can quickly and accurately predict instability timescales in compact multiplanet systems, and does so with an accurate uncertainty estimate for unfamiliar systems. This opens up the development of fast terrestrial planet formation models, and enables the efficient exploration of stable regions in parameter space for multiplanet systems.

The final growth of terrestrial bodies in current theories of planet formation occurs in a phase of giant impacts (1). During this stage, the number of planets slowly declines as bodies collide and merge (2, 3). Close planetary encounters and the wide dynamic range exhibited by the times between consecutive collisions computationally limit current numerical efforts to model this process. Two theoretical roadblocks impede the development of a more efficient iterative map for modeling planet formation. First, one must predict a distribution of instability times from a given initial orbital configuration. Second, one must predict a distribution of postcollision orbital architectures (e.g., ref. 4) subject to mass, energy, and angular momentum constraints. Toward this end, we focus on the long-standing former question of instability time prediction.

In the compact dynamical configurations that characterize the planet formation process, the simpler two-planet case is well understood analytically. In this limit, instabilities are driven by the interactions between nearby mean-motion resonances (MMRs), that is, integer commensurabilities between the orbital periods of the planets like the 3:2 MMR between Pluto and Neptune (5–8). While the general higher-multiplicity case is not yet understood, two important results guide our analysis and provide an important test for any model. First, when planets are initialized on circular orbits, chaos is driven by the overlap of three-body MMRs between trios of adjacent planets (9), and theoretical estimates of the timescale required for the orbits to reach orbit-crossing configurations accurately match numerical integrations (10). As we show below, such analytical estimates perform poorly in the generic eccentric case where the effects of two-body MMRs are dominant (10, 11). However, analytical and empirical studies agree that, while the dynamical behavior changes strongly from the two- to three-planet case (3, 12–18), three-planet systems are the simplest prototype for predictions at higher multiplicities in compact systems (10, 11).

We recently presented a machine learning model, dubbed the Stability of Planetary Orbital Configurations Klassifier, or SPOCK, trained to classify the stability of compact planetary systems over timescales of $10^{9}$ orbits (11). This represented a long-term effort to exploit the substantial but incomplete current analytical understanding (5, 6, 8, 19, 20) to engineer summary metrics that captured these systems’ chaotic dynamics; these features were then used by the machine learning model to classify whether the input configuration would be stable over $10^{9}$ orbits.

While simple binary stability classification is effective for constraining physical and orbital parameters consistent with long-term stability (21), other applications like modeling terrestrial planet formation require the prediction of continuous instability times. Additionally, several fields in which it is challenging to find effective handpicked features—such as computer vision, speech recognition, and text translation—have been revolutionized by neural networks in the last decade (notable early breakthroughs include refs. 22–24). Rather than relying on domain expert input, these flexible models learn data-driven features that can often significantly outperform human-engineered approaches. A key theme with deep learning models is that their structure resembles the hand-designed algorithm, but with added flexibility parametrized by neural networks (for discussion, see ref. 25). For example, modern computer vision models consist of learned convolutional filters which take the place of hand-designed filters in classic algorithms (26).

Pursuing a deep learning approach, we present a neural network that, trained only on short time series of the orbits in compact planetary systems, not only improves on long-term predictions of previous models based on engineered features (11, 27) but also significantly reduces the model bias and improves generalization beyond the training set. We design our model as a Bayesian neural network (BNN), which naturally incorporates confidence intervals into its instability time predictions, accounting for model uncertainty as well as the intrinsic uncertainty due to the chaotic dynamics. Finally, unlike previous machine learning models based on decision trees (11, 27), our model is differentiable. That is, we can extract from the model estimates of the derivatives of the predicted instability times with respect to the parameters defining the orbital configuration in question. Such gradient information can significantly speed up parameter estimation using Hamiltonian Monte Carlo techniques (28).

## Model

### Terminology.

In the interest of making this work accessible to readers from both the machine learning and natural science communities, here we give brief explanations for some terms used in machine learning research.

In machine learning, “regression” is a generic name for the problem of predicting a continuous variable from some input data. Iteratively optimizing the parameters of such a model is referred to as “training.” Modern machine learning often uses “neural networks”—which are universal function approximators that can be trained by gradient descent—for solving such regression problems on high-dimensional data. “Stochastic gradient descent” is when one uses only a fraction of data to estimate the gradient for each optimization step, and is an efficient strategy used to train neural networks. This gradient is taken of some “loss function” that one wishes to minimize—such as the mean-square error or the negative-log likelihood of the predictions with respect to some “targets.” Stochastic gradient descent is controlled by a “step size” parameter as well as the “batch size”—which controls how large a fraction of data are used to estimate the gradient. Parameters such as these optimization settings, as well as model specifications, are often referred to as “hyperparameters” to contextualize them relative to the “neural network parameters,” which are the actual parameters being optimized during training.

The “architecture” of a neural network refers to a specification of its layout and how each parameter is used. The most common type of architecture is a “multilayer perceptron”—or MLP—which is the core module that many deep neural networks are composed of. Internally, a neural network consists of “layers” of “learned features”—numbers that represent nonlinear combinations of the input data. Each layer of an MLP transforms an input vector by computing the product of a matrix of parameters with that vector, adding a vector of parameters, and then applying some nonlinear element-wise operation such as converting negative numbers to zero.

To protect against a potential scenario where a model simply memorizes the data, one partitions the data into “train/validation/test” subsets—the train part to be used for gradient descent, the validation part to be used for initial testing in order to tune hyperparameters, and the test part for evaluating the final performance of a model on unseen data.

### Dataset Generation.

We focus on the regime leading to typical compact multiplanet systems observed to date, with mass ratios with the central star ranging from $10^{-7}$ (roughly the ratio of the Moon-mass embryos thought to initially characterize the giant impact phase, relative to the Sun) to $10^{-4}$ (roughly Neptune’s mass relative to the Sun). As detailed in *Materials and Methods*, we place planets on nearly coplanar orbits, with adjacent planets spaced within 30 mutual Hill radii of one another (e.g., ref. 29).* Orbital eccentricities in observed systems are often poorly constrained, so we consider the range from initially circular to orbit-crossing values.

A central challenge is that the phase space is punctuated by narrow MMRs where instability times cannot only drop by orders of magnitude (30) but also can be stabilized inside small islands for particular combinations of masses, eccentricities, pericenter orientations, and orbital phase (31). In order to effectively sample these narrow regions where the dynamical behavior changes most strongly within our 21-dimensional phase space (a mass and six orbital elements for each planet), we train our model on the set of 113,543 publicly available, compact three-planet configurations in and near strong resonances, from ref. 11. In particular, this “resonant” dataset initializes one pair of planets in or near a strong MMR using analytical MMR models (20), while the third planet’s orbital parameters are chosen randomly. In *Results*, we test the model’s generalization to nonresonant systems.

Each initial condition was integrated for $10^{9}$ orbits of the innermost planet using the WHFast (fast Wisdom–Holman) integrator (32) in the REBOUND N-body package (33). If, at any point, two planets came within a distance of one another given by the sum of their Hill radii, the simulation was stopped, and the instability time was recorded. Because gravity is scale invariant, the instability time $t_{\text{inst}}$ is most usefully nondimensionalized by the innermost orbital period $P_{\text{orb}}$. Given the large dynamic range in timescales over which instabilities can occur, we define the dimensionless log instability time $T\equiv \log_{10}\left(t_{\text{inst}}/P_{\text{orb}}\right)$. Configurations with instability times longer than $10^{9}$ orbits (T>9) were labeled as stable, and integration was stopped.

### Network Architecture.

To predict systems’ instability times, we perform a short numerical integration of the first $10^{4}$ orbits, and use this time series to make long-term predictions. Each of the three planets’ three-dimensional (3D) positions and velocities correspond to six standard orbital elements (see *Materials and Methods* for details), which we record at $n_{t}=100$ equally spaced outputs across the short integration. In addition, we pass the three constant mass ratios for each planet relative to the star, as well as the time value, for a combined input matrix of real values $X\in R^{3+\left(19\times n_{t}\right)}$ for a given configuration.

Because the dynamics of compact multiplanet systems are chaotic, instability times for a given initial orbital configuration are effectively nondeterministic. Nevertheless, numerical experiments (34, 35) have shown that instability times for unstable, compact multiplanetary systems settle to well-defined, approximately log-normal distributions. Thus, rather than predicting a single instability time for a given orbital configuration, our model maps from an input initial orbital configuration to a predicted log-normal distribution of instability times, that is, a Gaussian distribution of T with mean μ and variance $\sigma^{2}$. This gives the network the flexibility both to model the fundamental uncertainties imposed by the chaotic dynamics and to incorporate model uncertainty into its predictions by assigning larger widths to configurations it is less sure about.

In our initial efforts, we experimented with various combinations of convolutional neural networks (see reviews by refs. 36–38), long short-term memory networks (39), 1D scattering transforms (40), regular MLPs (see ref. 25), and Gaussian processes (41). All of these models underperformed or tended to overfit the data.

**Fig. 1.**
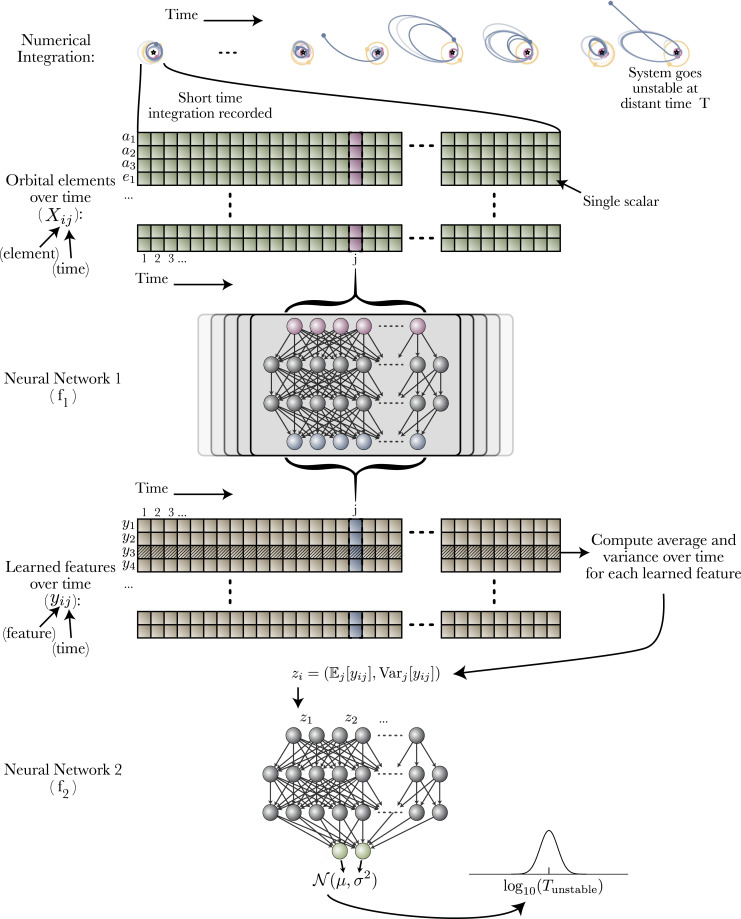
Schematic of our model. The model numerically integrates 10,000 orbits for a compact three-planet system (top) and records orbital elements at 100 times. Neural network $f_{1}$ creates learned summary features from these elements at each time. Neural network $f_{2}$ takes the average and variance of these features as input and estimates a distribution over possible instability times.

The fundamental challenge for making such predictions is the sharp transitions in dynamical behavior at MMRs, where instability times can change by several orders of magnitude (30) over subpercent changes in planetary orbital periods, that is, in the original space of orbital elements. We found substantially improved performance by structurally splitting the problem into three components: 1) Find a transformation from the sharply punctuated space of orbital elements to new variables. 2) Calculate statistical summaries of the time series in these transformed variables. 3) Use these summary features to predict a log-normal distribution over instability times for the input orbital configuration, parametrized by mean μ and variance $\sigma^{2}$. This is illustrated in Fig. 1.

We model steps 1 and 3 with neural networks $f_{1}$ and $f_{2}$, respectively. In step 2, we choose to use a well-motivated but nonstandard aggregation operation, and calculate both the mean and variance of each learned feature over time (deep neural networks traditionally use one of either sum, mean, or max for their internal aggregation). This structure was motivated by the hand-engineered approach of ref. 11, whose features are means and variances of theoretically motivated instability metrics. In this case, we give the machine learning model additional flexibility to learn its own instability metrics. This design process is analogous to how the invention of convolutional neural networks was motivated by creating similar structures to hand-engineered algorithms which convolve filters over an image.

### Likelihood.

Our model is parametrized by m=7,583 neural network parameters $\theta \equiv \left(\theta_{1},\theta_{2}\right),\theta \in R^{m}$ ($\theta_{1}$ for $f_{1}$ with 4,140 parameters, and $\theta_{2}$ for $f_{2}$ with 3,443 parameters). Defining the training set D as the collection of input orbital configurations and their associated N-body instability times, we seek the most likely set of model parameters given the data; that is, we maximize P(θ|D), which is, in turn, proportional to P(D|θ)P(θ).

Our model predicts a log-normal distribution of instability times for any input orbital configuration. For a given set of network parameters θ, the likelihood P(D|θ) is then simply the product of the probabilities that each training set example’s output $T_{i}$ is drawn from the associated Gaussian $N\left(\mu_{i},\sigma_{i}^{2}\right)$ predicted by the model. As discussed above, this choice is motivated by the numerical result that the distribution in T is normal, for different configurations with a wide range of mean instability times (35).

Note that we have 4<T≤9 as a constraint for unstable simulations: T<4 simulations are not included in the training set, and T>9 integrations were terminated at T=9 and have an unknown T. Thus, we build a truncated normal distribution with a cutoff at T=4, and with the cumulative probability of the Gaussian above T=9 being counted toward a classification of stability. A mathematical derivation of this likelihood is given in *Materials and Methods*.

P(θ) is a prior on the neural network’s parameters. The per-parameter prior here is unimportant: what matters is the prior induced on the output of the model, and we use an uninformative Gaussian prior on parameters to induce an uninformative prior on the output. See ref. 42 for a detailed discussion of priors in Bayesian deep learning.

### Bayesian Neural Network Implementation.

By having our model predict a distribution of instability times with a finite width, we account for intrinsic uncertainty (sometimes referred to as “aleatoric” uncertainty). However, we also wish to include extrapolation uncertainty (or “epistemic” uncertainty) for systems that differ from those found in the training set. To do this, we marginalize over potential model parameters, with what is referred to as a BNN. This is a neural network whose parameters are distributions rather than point values; the network is trained with Bayesian inference to estimate a posterior over the model’s parameters. To compute the prediction of such a network, one marginalizes over this parameter posterior, which naturally folds in extrapolation uncertainty.

This concept is familiar in traditional statistical inference, where one can marginalize out the internal nuisance parameters of a model using Markov chain Monte Carlo (MCMC) techniques. The fact that neural networks typically have millions of parameters renders MCMC computationally prohibitive, and various practical simplifications are adopted for implementing a BNN. The most common strategy is Monte Carlo dropout (43, 44) which treats the neural network’s parameters as independent Bernoulli random variables and has been used in several astronomical applications (45–48). A selection of other techniques includes Bayes by Backprop (49), Bayesian Layers (50), variants of normalizing flows (e.g., ref. 51), Bayes by Hypernet (52, 53), and many other strategies. One recently proposed strategy, named “MultiSWAG” (54, 55), learns a distribution over the posterior of parameters that best fit the training set, without a diagonal covariance assumption, and is much closer to standard MCMC inference. We experimented with a selection of common techniques—Monte Carlo dropout, Bayes by Backprop, and MultiSWAG—and found that “MultiSWAG” produced the best accuracy and uncertainty estimations on the validation dataset.

To move beyond a single best-fit set of parameters θ, SWAG, or “stochastic weight averaging Gaussian” (54, 56), instead fits a Gaussian to a mode of the posterior over θ (equivalently, one could say “a minima of the optimization surface”), with a low-rank approximation to the off-diagonal component of the covariance matrix. This was extended in ref. 55 to MultiSWAG, which repeats this process for several modes of the weight posterior, to help fill out the highly degenerate parameter space. This technique is summarized below.

1) Train $f_{1}$ and $f_{2}$ simultaneously via stochastic gradient descent until the parameters settle into a minimum of the weight posterior.
2) Increase the step size and continue training. This causes the optimizer to take a random walk in parameter space near the minima, which is assumed to look like a high-dimensional Gaussian.
3 Accumulate the average parameters along this random walk as well as each parameter’s variance, and a low-rank approximation of the off-diagonal covariance matrix. The definition of this matrix is detailed in ref. 55; it attempts to approximate the overall shape (i.e., the principal components) of the region around the minima.
4) The average parameters not only provide better generalization performance (stochastic weight averaging or SWA), but we have additionally fit a Gaussian to a mode of the parameter posterior. We can thus sample parameters from this Gaussian to marginalize over parameters. This is SWAG (54).
5) The next step is to repeat this entire process from a different random initialization of the parameters. This will find another mode of the parameter posterior.
6) Fit ∼30 different modes. We can then sample parameters from different modes in the parameter posterior, which gives us a more rigorous uncertainty estimate. This is MultiSWAG (55).

Using stochastic gradient descent on a neural network’s parameters is related to MCMC sampling the parameter posterior (57, 58), so this aforementioned process allows one to learn a Bayesian posterior over the parameters of a neural network. We call this learned distribution over the parameters $P_{\text{MultiSWAG}}\left(\theta \right)$.

Once we have learned $P_{\text{MultiSWAG}}\left(\theta \right)$, we can draw from it—by picking a random mode, and sampling a Gaussian according to the learned covariance and mean—to sample a set of network parameters θ. Given some input data and a draw of the model’s parameters, the model then predicts a log-normal distribution of instability times with mean μ and variance $\sigma^{2}$ for the given input orbital configuration, from which we can sample a log instability time *T*. We can write a forward model for this prediction as follows:

$$\text{Sample model parameters.}\;\left(\theta_{1},\theta_{2}\right)∼P_{\text{MultiSWAG}}\left(\theta \right),$$ [1]

$$\text{Compute learned features.}\;\begin{array}{c} y_{t}=f_{1}\left(x_{t};\theta_{1}\right)\\ \;\text{for each}x_{t}\equiv X_{:,t}, \end{array}$$ [2]

$$\text{Aggregate learned features.}\;z∼\left(E_{t}\left[y_{t}\right],{\text{Var}}_{t}\left[y_{t}\right]\right),$$ [3]

$$\text{Predict time distribution.}\;\left(\mu ,\sigma^{2}\right)=f_{2}\left(z;\theta_{2}\right),$$ [4]

$$\text{Sample instability time.}\begin{array}{c} T_{\text{instability}}=10^{T}\\ \;\text{for}T∼N\left(\mu ,\sigma^{2}\right), \end{array}$$ [5]

where t is a time step from 1 to 100. Here, we have labeled $y_{t}$ as the learned transformed variables for a single time step of the system (brown cells in Fig. 1), and z as the average and variance of these transformed variables over time. To account for statistical errors due to our finite number of time series samples, we sample the z from normal distributions with frequentist estimates of the variance in the sample mean and variance: ${\text{Var}}_{t}\left[y_{t}\right]/n_{t}$ and $2{\text{Var}}_{t}{\left[y_{t}\right]}^{2}/\left(n_{t}-1\right)$, respectively. A Bayesian graphical model for this is shown in *Materials and Methods*. Repeatedly sampling in this way—drawing a set of parameters, computing a prediction, and sampling a time—provides a predicted distribution of *T* given the input orbital configuration, marginalized over the posterior distribution of network parameters θ.

We split our data into 60/20/20% train/validation/test, train our model on 60% of our ≈100,000 training examples of resonant and near-resonant systems, and validate it on half of the remaining data to tune the hyperparameters. Hyperparameters for our model are given in *Materials and Methods*, and we also release the code to train and evaluate our model.

With this trained model, we then explore its performance on the remaining 20% holdout data from the resonant dataset, as well as other datasets described below.

## Results

### Resonant Test Dataset.

For a given orbital configuration, our probabilistic model produces one sample of T. If a given sample is above T=9, we treat the sample as a “stable” prediction. Since we are unable to make specific time predictions above the maximum integration time in our training dataset of T=9, we resample from a user-defined prior P(T|T≥9) for each occurrence. For the purposes of this study, we assume a simple analytic form for this prior, although follow-up work on this prior is ongoing (see *Materials and Methods*).

For all results, we sample 10,000 predicted values of the posterior over T per planetary system. We compare our predictions against several alternatives which are explained below. Since the models we compare against can only produce point estimates, while our model predicts a distribution, we take the median of our model’s predicted posterior over T. This is used for plotting points, as well as for computing root-mean-square prediction errors.

**Fig. 2.**
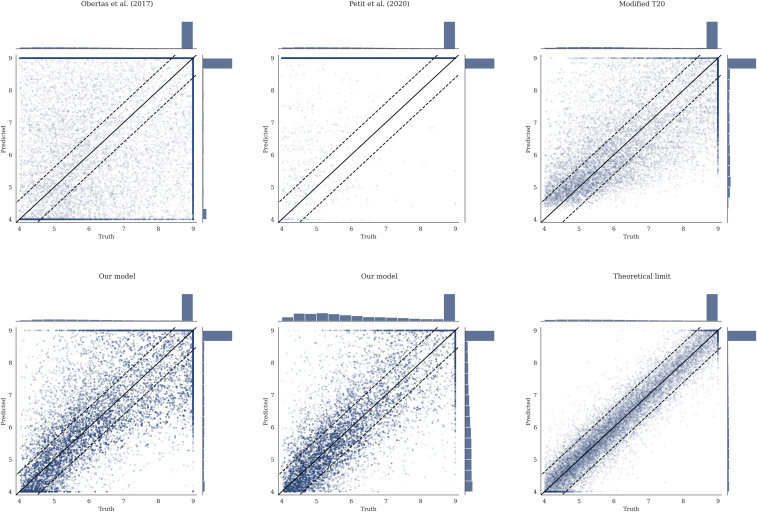
Density plots showing the predicted versus true instability times for various models on the random dataset. All predictions outside of T∈[4,9] are moved to the edge of the range. For *Bottom Left* and *Bottom Middle*, which show the predictions of our model, transparency shows relative model-predicted SNR. The theoretical limit, using the numerical experiments of ref. 35, is given in *Bottom Right*. The 0.43 dex RMSE average from this is used to give the dotted contours in all plots. *Top Left* shows the predictions of ref. 30, *Top Middle* shows the predictions of ref. 10, and *Top Right* shows the predictions of ref. 11.

We first compute the N-body versus predicted (median) T value over the holdout test dataset of ≈20,000 examples not seen during training, which can be seen in Fig. 2, *Bottom Middle*. We reiterate that the N-body instability times measured for the various orbital configurations in our training set are not “true” instability times but rather represent single draws from the different planetary systems’ respective instability time distributions, established by their chaotic dynamics. To estimate a theoretical limit (Fig. 2, *Bottom Right*), we use the results from ref. 35, who find that the *T* values measured by N-body integrations (*x* axis of Fig. 2) should be approximately normally distributed around the mean instability time predicted by an ideal model. We use a random SD drawn from the values measured empirically for compact systems by ref. 35, which they find are sharply peaked around ≈0.43 dex, independent of whether or not the system is near MMRs, and valid across a wide range of mean instability times. We plot this representative intrinsic width of 0.43 dex as dotted lines in Fig. 2 for comparison.

While we defer a detailed comparison to previous work to the following section, we measure an RMS error (RMSE) of 1.02 dex for our model on the holdout test set. We note that, while the RMSE is an intuitive metric for comparing models, it does not provide a full picture for a model that is trained on a different loss function to predict both μ and $\sigma^{2}$. A model that can predict its own $\sigma^{2}$ will sacrifice worse μ accuracy in challenging regions of parameter space to better predict it on more easily predictable configurations. For comparison, if we weight the RMSE by the predicted signal-to-noise ratio (SNR), $\mu^{2}/\sigma^{2}$, the model achieves 0.87 dex, within a factor of ≈2 of the theoretical limit. These uncertainties provide confidence estimates in the predicted values, and can indicate to a user when to invest in a computationally costly direct integration. We apply transparency to our predictions in Fig. 2 according to the model-predicted SNR, highlighting that the poorest predictions were typically deemed uncertain by the model.

**Fig. 3.**
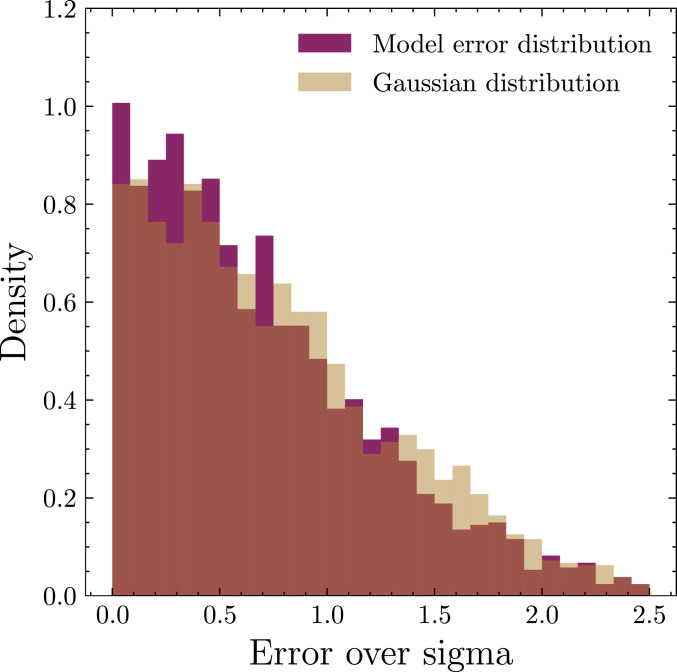
A histogram of our model’s error distribution—the difference between predicted and true instability time, divided by the model’s predicted σ—compared with a histogram of samples of a Gaussian. This plot demonstrates that, were our model to predict some μ,σ, one would expect 68% of the true instability time values to fall within μ±1σ, as expected for a true Gaussian distribution.

We also quantitatively test whether the model-predicted uncertainties σ accurately capture the spread of N-body times around the predicted mean values μ. For each test configuration, we predict μ, subtract it from its respective T measured by N-body integration, and divide by the predicted σ. If this distribution approximates a Gaussian distribution of zero mean and unit variance, the model’s uncertainty estimates are accurate. We find that a Kolmogorov−Smirnov test cannot confidently distinguish our predictions from this ideal Gaussian (*P* value of 0.056), and we plot the two distributions in Fig. 3.

Finally, we note that not all of the input orbital elements to the model are independent. For example, only the ratios of semimajor axes are dynamically relevant, which is two rather than three variables. In addition, the rotational invariance of this problem implies that only differences between the ascending node longitudes, and not their individual values, are physically relevant. We can use this rotational invariance as a test case: If we pass our trained model the default SPOCK test configuration, and repeat with an example rotated by π about the z axis, we find that a two-sample Kolmogorov−Smirnov test cannot confidently distinguish between the distributions of 1,000 samples each (*P* value of 0.30). The model has therefore seemed to learn an approximate rotational invariance in the problem directly from the training set.

### Comparison to Previous Work.

Guided by the dynamical intuition that short-timescale instabilities are driven by the interaction of MMRs (5, 8, 11), we chose to train our model on systems with particular period ratios and orbital elements in the narrow ranges near such resonances where the dynamical behavior changes sharply (30). It is therefore important to test how well such a model generalizes to a more uniform coverage of parameter space, given that most observed orbital architectures are not in MMRs (possibly because such configurations typically have short lifetimes and have been eliminated). Additionally, previous work has typically ignored the sharp variations near MMRs to fit overall trends in instability times (30), so a test on resonant systems would not provide a fair comparison.

For this generalization test and comparison, we use the “random” dataset of ref. 11, 25,000 three-planet systems with the same mass ratio and inclination distributions as above, and eccentricities drawn log uniformly from $\approx 10^{-3}$ to orbit crossing. Note that we do not train our model on this dataset; we only use it for testing. Rather than drawing near-integer period ratios as in our resonant training set, the spacing between adjacent planets is drawn uniformly between [3.5, 30] mutual Hill radii (see ref. 11).

We find that our model exhibits only a minor loss in performance (1.20 vs. 1.02 dex RMSE) generalizing to this uniform distribution of orbital configurations (Table 1). This supports the assertion that instabilities in compact systems within $10^{9}$ orbits are dominantly driven by the MMRs we focused on in our training sample (11). To compare our results to the extensive set of past efforts, we divide previous approaches into three broad groups.

**Table 1.** Statistical summaries of each estimator applied to a holdout test portion of the resonant dataset, and to all of the random dataset

Model	RMSE	Classif. accur.	Bias* for T∈ (4, 5)	Bias for T∈ (8, 9)
Resonant				
Obertas et al. (2017) (30)	2.12	0.628	1.04	−1.71
Petit et al. (2020) (10)	3.22	0.530	3.99	0.54
Tamayo et al. (2020) (27)	1.48	0.946	2.07	−0.62
Modified^†^ Tamayo+20	0.99	0.946	0.65	−0.60
Ours	1.02	0.952	0.29	−0.38
Ours, SNR-weighted	0.87	0.971	0.18	−0.25
Theoretical limit	0.43	0.992	0.05	−0.04
Random				
Obertas et al. (2017) (30)	2.41	0.721	2.15	−0.93
Petit et al. (2020) (10)	3.09	0.517	4.17	0.50
Tamayo et al. (2020) (27)	1.24	0.949	1.16	−0.59
Modified^†^ Tamayo+20	1.14	0.945	0.79	−0.70
Ours	1.20	0.939	0.40	−0.51
Ours, SNR-weighted	1.09	0.959	0.23	−0.49
Theoretical limit	0.44	0.989	0.06	−0.04
	(dex)	(AUC)	(dex)	(dex)

See *Theoretical Limit* section of *Materials* for details. Classif. accur. refers to the classification accuracy. See *Comparison to Previous Work* for details.

*Average difference between predicted minus true T in given range.

^†^Modified and retrained for regression.

First, many authors have run N-body integrations along low-dimensional cuts through the parameter space of compact orbital configurations, and fit simple functional forms to the resulting trends in instability times. For example, several studies have highlighted the steep dependence on interplanetary separation, while fixing orbits to be coplanar and initially circular, and planets to be equal mass and equally separated from one another (12, 14, 16, 17, 30). We compare the performance of the fit from the study in ref. 30, using five equally spaced Earth-mass planets (mass ratio $\approx 3\times 10^{-6}$) on our random test set in Fig. 2, *Top Left*, with a resulting RMSE of 2.41 (we also test our model on the simulations used in ref. 30). Follow-up studies have incorporated the effect of finite inclinations and eccentricities (13, 15, 59, 60), but they consider equal initial eccentricities, planetary masses, etc., in order to fit simple models. We conclude that, while such controlled experiments yield insight into the underlying dynamics (9, 15, 61), instability times depend sensitively on masses and several orbital parameters, rendering empirical fits to low-dimensional cuts in the parameter space of limited applicability.

Second, previous authors have developed analytical instability time estimates from first principles. These have been most successful in the limit of initially circular orbits, where three-body MMRs have been identified as the dominant driver of chaos (9). Recent work (10) has extended this theory to provide accurate instability time estimates. We will compare the predictions of our model to this limit of initially circular orbits, in the next section. Here we simply emphasize the point by ref. 10 that such predictions perform poorly at finite eccentricities (Fig. 2, *Top Middle*), likely due to the dominant effects of stronger two-body MMRs. The fact that the analytic model predicts the vast majority of systems to be stable implies that most of our test configurations would be stable on circular orbits, but that finite orbital eccentricities strongly modulate instability times.

The final approach is to make predictions across the high-dimensional space of orbital configurations using machine learning (11, 27). We consider two variants of ref. 11 adapted for regression. The first, labeled “Tamayo et al. (2020)” in Fig. 2, is to simply use model identical to that of ref. 11 but map the probability estimates of stability past $10^{9}$ orbits through an inverse cumulative distribution of a log-normal with an optimized constant SD. For the second, labeled “Modified Tamayo+20,” the model is an XGBoost (62) regression model (rather than classification) retrained on the same features as used in ref. 11.

We find that our model achieves similar performance to the Modified Tamayo+20 variant (Fig. 2 , *Top Right* and Table 1), although the latter exhibits significant bias. We quantify this bias for each model in the range T∈ (4, 5) and T∈(8, 9). As is evident in Table 1 as well as Fig. 2, the model introduced in this work exhibits significantly reduced bias compared to other models. Including SNR weighting further reduces bias. Bias is a measure of the generalizability of a model to out-of-distribution data (see chapter 7 of ref. 63), and so is an important metric for understanding how these predictive models will extrapolate to new data. Our model achieves predictions that are more than two orders of magnitude more accurate than the analytic models in each case, for example, $10^{2.41/1.09}\approx 162\times$ when comparing our SNR-weighted model with ref. 30 on the random dataset.

Finally, we can make a comparison to the original classification model of ref. 11 by using our regression model as a form of classifier. We count the fraction of samples above T=9 as the probability a given system is stable, and measure the performance of the classifier with AUC (area under curve) for the receiver operating characteristic curve—a classification metric with 1 indicating a perfect model and 0.5 indicating random guesses) for a range of threshold probabilities for stability (Table 1).

### Five-Planet Generalization with Comparison.

As a second generalization test of our model, we compare its performance on the limiting case considered by ref. 30. This case of five equally spaced, Earth-mass planets on initially circular and coplanar orbits differs significantly from our training set of resonant and near-resonant, eccentric, and inclined configurations of three planets with unequal masses. This dataset contains 17,500 simulations numerically integrated for $10^{10}$ orbits (30). This generalization to a limiting set of higher-multiplicity configurations provides a stringent test of whether the model has learned features of the dynamics or whether it is naively interpolating across the distribution of orbital configurations present in our training dataset.

To extend our three-planet predictions to higher multiplicity systems, we perform the same short integration for all planets, but pass time series for each adjacent trio of planets to the model separately. The model samples a single instability time for each adjacent trio, and the minimum across this set is adopted as the instability time for the system, as an estimate of the time for the first trio to go unstable. This procedure is then repeated, and we record the median and CIs of the resultant distribution in T. Such a reduction of compact multiplanet systems to sets of adjacent trios has been proposed on theoretical (9, 10) as well as empirical (11) grounds. This is motivated by the fact that the perturbative effects of planets on one another fall off exponentially with separation (9, 10), so nonadjacent interactions can largely be ignored.

**Fig. 4.**
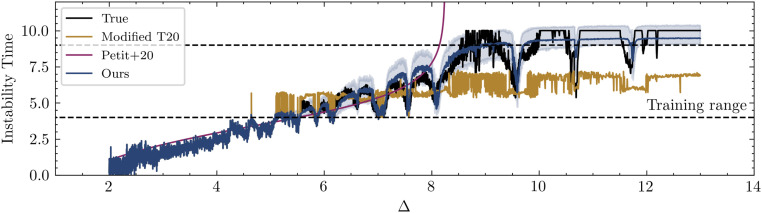
The median instability time predictions of our model for the five-planet systems used in ref. 30. These systems have fixed interplanetary separation between adjacent orbits, which is labeled on the *x* axis. Error bars fill out the 68% CI. The predictions from refs. 10 and 11 are overplotted. Residuals are shown in *Materials and Methods*.

**Fig. 5.**
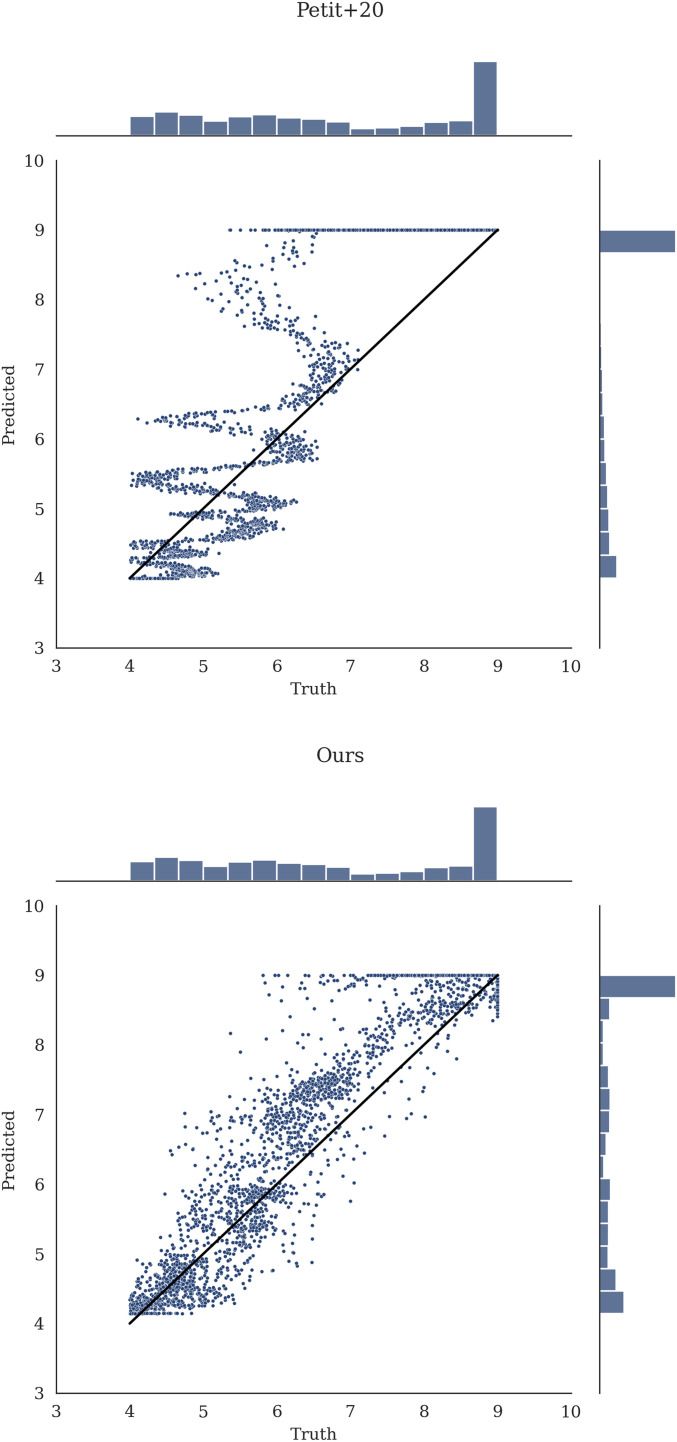
Residuals for predictions of instability time on the five-planet dataset in Fig. 4, using data from ref. 30.

The predictions can be seen in Fig. 4 and are remarkably accurate, despite our model never seeing a system with five planets during training. We overplot the analytical result of ref. 10, in magenta, developed from first principles for such cases with initially circular orbits, including a manual correction for five-planet systems. Our model captures the same overall trend, but additionally identifies the various dips, which correspond to locations of different MMRs (30). We emphasize that our model was trained on the general eccentric case where the magenta model of ref. 10 does not apply (Fig. 2), yet the generalization to this limiting case is excellent. In addition to matching the overall trend of ref. 10, our model captures the additional instability time modulations at MMRs, as can be seen more clearly in the residuals in Fig. 5. Additionally, our model generalizes much better than the predictions of the modified regression model based on ref. 11 based on manually engineered features (gold in Fig. 4).

### Interpretation.

In industry, machine learning is used to make predictions as accurate as possible, even at the expense of a more interpretable model. However, in physics, we are fundamentally interested in understanding problems from first principles.

**Fig. 6.**
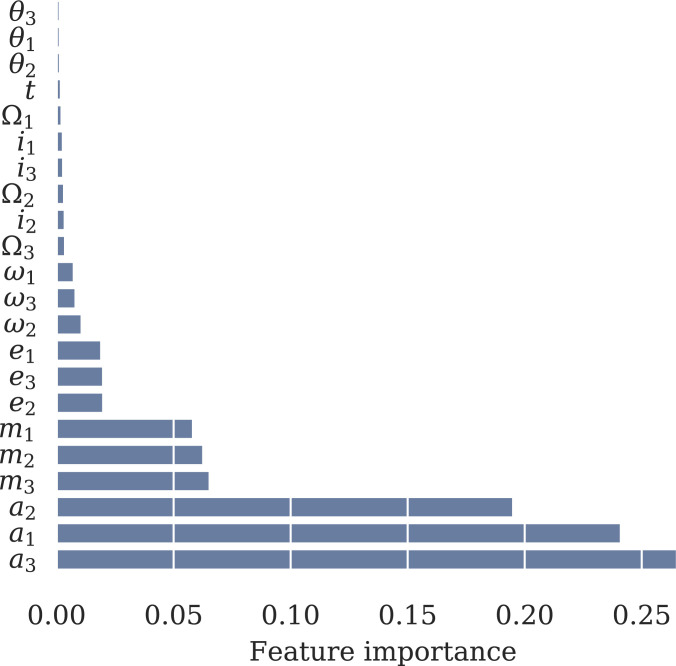
Feature importances in the model, calculated as the root-mean-square of the gradients of the model’s output with respect to the input features, and normalized.

Obtaining such an explicit interpretation of our model will be difficult. However, as a first step, we consider the feature importances of our model: What orbital elements is it using to make predictions, and does this align with expectations? To do this feature analysis, we find the “saliency map” of the model (25), which we compute as the variance of the gradient of the predicted μ value with respect to the input orbital elements. This gives us a multidimensional array over feature, simulation, time step, and model, representing how much the predicted μ value will change should that feature be infinitesimally increased. We compute the variance of the gradients over time and each simulation, and then average these variances over sampled network parameters θ. This gives us a rough estimate for the importance of each feature, which we visualize in Fig. 6.

To compare these importances to other work (11), we argue empirically that the short-timescale instabilities we probe here in compact systems are driven specifically by the interactions between MMRs. A classical result of celestial mechanics is that, in the absence of such MMRs, the long-term dynamics keeps the semimajor axes fixed. Variations in the semimajor axes during the short integrations thus act as a proxy for the importance of nearby MMRs (27), and we see that, indeed, the semimajor axes exhibit the highest feature importance in our model Fig. 6. Note that $a_{1}$ is normalized as $\left(a_{1}\left(t\right)/a_{1}\left(0\right)\right)$ (*Materials and Methods*), so the feature importance signifies that deviations in $a_{1}$ from the initial value of one are evidently important to the model. The fact that the model ascribes comparable feature importance to any given orbital element for each of the three planets also suggests a physically reasonable model.

We note that there is a small but nonzero significance of the instantaneous feature for time. This can be interpreted as being important because the model takes the first $10^{4}$ orbits as input, and can predict instability for the system as low as $10^{4.1}$. Thus, the orbital parameters given at $10^{4}$ orbits may be more important than the orbital parameters at $10^{0}$ orbits for predicting such unstable systems, and thus the time feature is used. The time feature would be less important for a system that goes unstable near $10^{9}$ orbits, as the relative importance of the system’s parameters at $10^{4}$ orbits is comparable to that at $10^{0}$ orbit.

Because we chose to structure our model to take means and variances of the times series of the learned features, it may be possible to extract explicit interpretations of our model via symbolic regression. Given that our approach is structurally similar to that of a graph neural network (64), the frameworks of refs. 65–67 would be particularly applicable. This would be done by finding analytic forms for $f_{1}$, representing each of the transformed variables, and then finding an analytic form for $f_{2}$, to compute the instability time given the transformed variables. This type of explicit analysis of the model will be considered in future work. This technique is not immediately applicable, as it requires a specific regularization on the training which is incompatible with MultiSWAG.

## Conclusion

We have described a probabilistic machine learning model—a BNN—that can accurately predict a distribution over possible instability times for a given compact multiplanet exoplanet system. Our model is trained on the raw orbital parameters of a multiplanet system over a short integration, and learns its own instability metrics. This is contrasted by previous machine learning approaches which have given their models hand-designed instability metrics based on specialized domain knowledge.

Our model is more than two orders of magnitude more accurate at predicting instability times than analytical estimators, while also reducing the bias of existing learned models by nearly a factor of 3. We also demonstrate that our model generalizes robustly to five-planet configurations effectively drawn from a 1D cut through the broad parameter space used to train the model. This improves on the estimates of analytic and other learned models, despite our model only being trained on compact three-planet systems.

Our model’s computational speedup over N-body integrations by a factor of up to $10^{5}$ enables a broad range of applications, such as using stability constraints to rule out unphysical configurations and constrain orbital parameters (21), and to develop efficient terrestrial planet formation models. Toward this end, our model will be made publicly available through the SPOCK package, with training code also available in a separate git repository.^†^

## Materials and Methods

### Parameters.

A planetary orbit can be described with six coordinates at any given time. We choose to use eccentricity (e), semimajor axis (a, normalized to the initial innermost planet’s value: i.e., $a_{1}\left(t\right)/a_{1}\left(0\right),a_{2}\left(t\right)/a_{1}\left(0\right),\ldots$), inclination (i), longitude of the ascending node (Ω), longitude of pericenter (ϖ), and true longitude (θ). We also pass the mass of each planet normalized to the star’s mass to each call of $f_{1}$ in Eq. **2**. For each of the angular coordinates excluding inclination, we split into two values—one for the sine and one for the cosine of the value—before passing to the first neural network.

### Likelihoods.

Here we give a mathematical derivation of the likelihood used to train our model. Our goal is to estimate the distribution P(T|X), for time series data $X\in R^{3+19n_{t}}$. We construct a probabilistic model defined by Eqs. **1**–**5**, with parameters $\theta \in R^{m}$, where m=7,583 is the total number of parameters, which takes a time series for three planets, X, and produces a normal probability distribution over T, parametrized by two scalars: $\left(\mu_{\theta }\left(X\right),\sigma_{\theta }^{2}\left(X\right)\right)$. $\mu_{\theta }$ is the center of the instability time, and $\sigma_{\theta }$ is the SD in that estimate. The distribution over T parametrized by the model is equal to

$$P\left(T|X,\theta \right)=\left\{\begin{array}{cc} \frac{A\left(\mu_{\theta }\left(X\right),\sigma_{\theta }^{2}\left(X\right)\right)}{\sqrt{2\pi }\sigma_{\theta }\left(X\right)}\exp\left(-\frac{{\left(T-\mu_{\theta }\left(X\right)\right)}^{2}}{2\sigma_{\theta }{\left(X\right)}^{2}}\right),\; & T<9\\ \frac{1}{2}\left(1+\text{erf}\left(\frac{\mu_{\theta }\left(X\right)-9}{\sqrt{2}\sigma_{\theta }\left(X\right)}\right)\right)P\left(T|T\geq 9\right),\; & T\geq 9. \end{array}\right.$$ [6]

This distribution is motivated by two things. First, as in ref. 35, exoplanet instability times usually follow a normal distribution in logarithmic instability time, regardless of how large this time is. Therefore, we predict a normal distribution in T for times under T<9. Second, due to computational costs, we only simulate systems up to $10^{9}$ orbits; hence we use a model that is independent of P(T|T≥9). We calculate the cumulative probability of the normal distribution falling T≥9 to calculate the probability of the value being stable. Here, A is a normalization function from the fact that we cut off the probability at T=4. Thus,

$$A\left(\mu_{\theta }\left(X\right),\sigma_{\theta }^{2}\left(X\right)\right)=\frac{2}{1+\text{erf}\left(\frac{\mu_{\theta }\left(X\right)-4}{\sqrt{2\sigma_{\theta }^{2}\left(X\right)}}\right)}.$$ [7]

This term, from our prior that T>4, helps remove bias from our model, as can be seen in Fig. 2. Without this term in the model, we would be artificially punishing predictions at low T values.

**Fig. 7.**
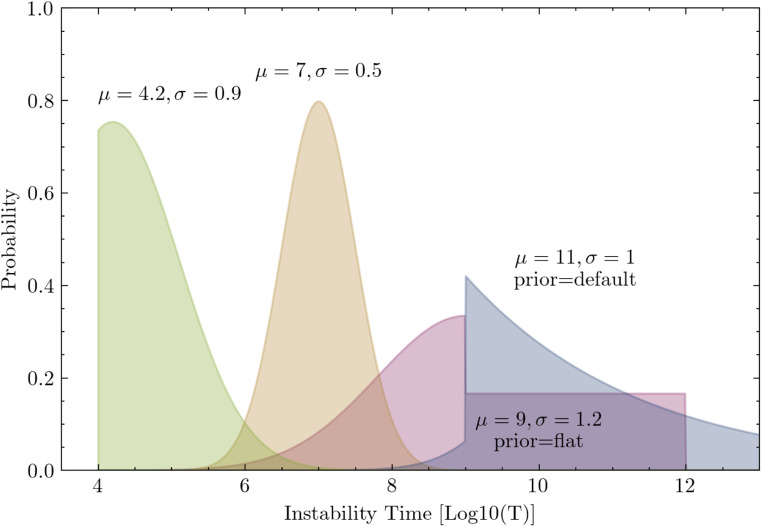
Example likelihoods for various choices of $\mu ,\sigma^{2}$ corresponding to Eq. **6**. For configurations stable past $10^{9}$ orbits, we visualize some example priors that one might select for inference, although we note that the choice of this prior does not affect the training of our model.

Assuming we produce a point-wise dataset $D={\left\{\left(T_{i},X_{i}\right)\right\}}_{i=1:N}$ via numerical integration, where $T_{i}\equiv 9$ indicates that the system is stable beyond $10^{9}$ orbits, the log-likelihood for this model is equal to

$$\log P\left(D|\theta \right)\propto \underset{i}{\sum }\left\{\begin{array}{cc} -\frac{{\left(T_{i}-\mu_{\theta }\left(X_{i}\right)\right)}^{2}}{2\sigma_{\theta }{\left(X_{i}\right)}^{2}}-\log\left(\sigma_{\theta }\left(X_{i}\right)\right)\;\\ \;-\log\left(1+\text{erf}\left(\frac{\mu_{\theta }\left(X_{i}\right)-4}{\sqrt{2\sigma_{\theta }^{2}\left(X_{i}\right)}}\right)\right),\; & T_{i}<9\\ \log\left(1+\text{erf}\left(\frac{\mu_{\theta }\left(X_{i}\right)-9}{\sqrt{2\sigma_{\theta }^{2}\left(X_{i}\right)}}\right)\right),\; & T_{i}\equiv 9, \end{array}\right.$$ [8]

assuming a fixed prior P(T|T≥9). Note how this decouples the loss for the stable values $T_{i}\geq 9$ from the prior P(T|T≥9), meaning the choice of prior will have no effect on our model, and can be chosen by a user after training. Examples of this are plotted in Fig. 7. Now, we also marginalize the model parameters θ, to incorporate epistemic uncertainty, and account for model biases due to the random seed used in initializing and training the model. We proceed as follows:

P(T|X,D)∝∫P(T,θ|X,D)dθ [9]

∝∫P(T|X,D,θ)P(θ|X,D)dθ [10]

∝∫P(T|X,θ)P(θ|D)dθ. [11]

**Fig. 8.**
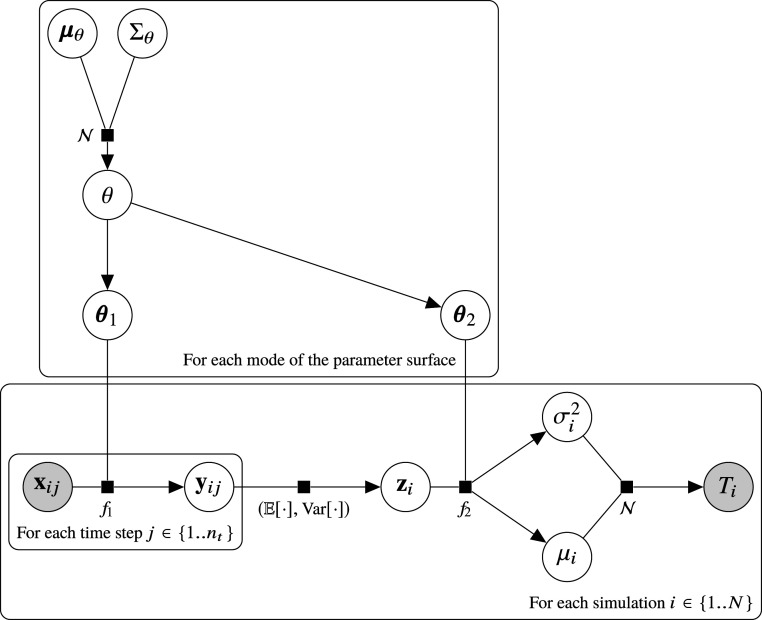
Bayesian graphical model representing our inference scheme for the instability time with BNNs, which goes from time series created via short-term integration (${\left\{x_{ij}\right\}}_{j=\left[1:n_{t}\right]}$) to a prediction of a (logarithmic) instability time ($T_{i}$) for each simulation i. The $f_{1}$ and $f_{2}$ are neural networks parametrized by $\theta \equiv \left(\theta_{1},\theta_{2}\right)$. A distribution over θ is learned according to the likelihood $P\left(\theta \right)\prod_{i}P\left(T_{i}|{\left\{x_{ij}\right\}}_{j};\theta \right)$. The model is given in Eqs. **1**–**5**. Notation follows that of ref. 68. Vectors are bolded, and matrices are capitalized.

We first maximize the likelihood of the model, to find P(θ|D). We factor the joint probability using Fig. 8, and proceed as follows:

$$P\left(\theta ,D\right)\propto P\left(\theta \right)\underset{i}{\prod }\int P\left(X_{i}\right)P\left(T_{i}|\mu_{i},\sigma_{i}^{2}\right)$$ [12]

$$\times P\left(\mu_{i},\sigma_{i}^{2}|\theta ,X_{i}\right)d\mu_{i}d\sigma_{i}^{2}$$ [13]

$$\text{Using}P\left(\theta |D\right)\propto \frac{P\left(\theta ,D\right)}{P\left(D\right)}$$ [14]

$$\Rightarrow P\left(\theta |D\right)\propto P\left(\theta \right)\underset{i}{\prod }\int P\left(T_{i}|\mu_{i},\sigma_{i}^{2}\right)P\left(\mu_{i},\sigma_{i}^{2}|\theta ,X_{i}\right)d\mu_{i}d\sigma_{i}^{2},$$ [15]

where $P\left(T_{i}|\mu_{i},\sigma_{i}^{2}\right)$ is given by Eq. **6** as the log-likelihood for our model, and $P\left(\mu_{i},\sigma_{i}^{2}|\theta ,X_{i}\right)d\mu_{i}d\sigma_{i}^{2}$ is our forward model given Eqs. **2**–**5**. Finally, we can write down the loss of our model, our function to minimize, as the negative logarithm of Eq. **15**, as follows:

$$\text{Loss}\left(\theta \right)=-\log\left(P\left(\theta \right)\right)-\underset{i}{\sum }E_{\left(\mu_{i},\sigma_{i}^{2}\right)\approx f\left(X_{i};\theta \right)}\log\left(P\left(T_{i}|\mu_{i},\sigma_{i}^{2}\right)\right),$$ [16]

where $f\left(X_{i};\theta \right)$ is the combined model Eqs. **2**–**5** for a given θ, and E is used to refer to the fact that z is sampled in Eq. **3**, so we average the loss over samples. We set P(θ) equal to a zero-centered uninformative Gaussian prior over the parameters. If this were a neural density estimator instead of a full BNN, we would minimize this for a single value of θ. Alternatively, we can sample θ≈P(θ|D) with a BNN algorithm. We use the MultiSWAG algorithm to do this (55), as described in *Bayesian Neural Network Implementation*, and aim to estimate the true parameter posterior P(θ|D) with our learned distribution $P_{\text{MultiSWAG}}\left(\theta \right)$.

### Training.

We have $n_{t}=100$ uniformly spaced samples of our integration over 10,000 initial orbits (the unit orbit is the initial period of the innermost planet). During training, we randomly select between 5 and 100 time steps, with replacement, to feed to the model. This is a type of data augmentation that improves generalization of the model. Since we are working with a varying number of orbit samples, we also sample the averages and variances from Gaussians over their frequentist distributions: $\mu \pm {\text{Var}}_{t}\left[y_{t}\right]/n_{t}$ for the mean, and $\sigma^{2}\pm 2{\text{Var}}_{t}{\left[y_{t}\right]}^{2}/\left(n_{t}-1\right)$ for the variance, where $n_{t}$ is the number of samples, and ${\text{Var}}_{t}$ is the sample variance. This will naturally allow the model to grow increasingly certain if a longer time series is given as input, since the averages and variances of the transformed coordinates are less subject to small-sample uncertainty.

### Hyperparameters.

Here we give a technical description of our neural network hyperparameters with terms used in machine learning literature. For our final model, we set both $f_{1}$ and $f_{2}$ to be MLPs with rectified linear unit (ReLU) activations: one hidden layer and 40 hidden nodes each (i.e., a matrix multiplication, a ReLU, a matrix multiplication, a ReLU, and another matrix multiplication). The number of calculated transformed features from $f_{1}$ is 20, meaning $f_{2}$ takes 40 features as input. We take 500,000 stochastic gradient descent optimization steps with random batches of simulations with a batch size of 2,000 with a cosine-annealed step size (69, 70) from $5\times 10^{-4}$ down to $5\times 10^{-8}$. This is followed by 50,000 additional steps at a fixed step size (presumably within a mode of the posterior) of $2.5\times 10^{-4}$, to record points of a Gaussian mode on the weight posterior. Gradient clipping on the $L_{2}$ norm of the gradients is used, with a clipping value of 0.1. A small amount of noise is added to the input features and summary statistics to encourage the model to only use features that are useful: A Kullback−Leibler divergence loss term is added to the loss function on this noise, with a multiplier of $10^{-5}$ on the input and $10^{-3}$ on the summary. This noise is not added during evaluation, only during training. We choose five as the minimum number of time steps to pass to the model. We rescale the data to have a zero mean and unit variance for each feature (i.e., we normalize with a mean and variance calculated over the entire training set and time series). All of these parameters were found with the hyperopt package with a 20% validation set, with a smaller number of training steps and accelerated step size schedule.^‡^Finally, we train an ensemble of 30 independent models, which represents 30 modes of the weight posterior. Each stored model contains the mean parameters, the mean square of the parameters, and a matrix of 30 (K=30) recorded deviations from the mean parameters, which represent the off-diagonal covariance. In total, this results in 30×32×7,583=7,279,680 saved parameters for describing the full MultiSWAG distribution. For a complete technical description of these details, our full training code is available at https://github.com/MilesCranmer/bnn_chaos_model.

### Approximating the Cumulative Distribution of a Gaussian.

**Fig. 9.**
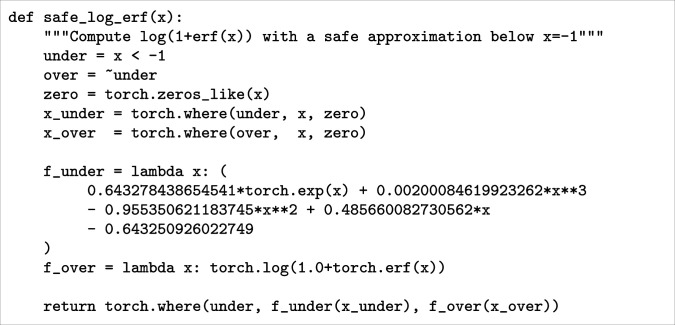
A differentiable torch implementation of log(1+erf(x)) which uses an analytic approximation for x<−1.

Due to precision issues of 32-bit floating point numbers, our autodifferentiation software, torch (71), is incapable of accurately calculating log(1+erf(x)) and its gradients as x decreases below −1. Because we heavily rely on this function in our log-likelihood for training our model, and need to pass gradients through it, we needed to approximate it for large negative x values. Otherwise, we found that the gradients in our model would often approach very large values, and training would fail. We approximate this function with an analytic continuation via symbolic regression using PySR (Python Symbolic Regression) (67). We generate values of this function in high-precision code, and then fit analytical forms with PySR. We find that the function is very accurately approximated over x∈[−5,−1] by

$$\begin{array}{ll} \log\left(1+\text{erf}\left(x\right)\right) & \approx 0.64325+0.48566x-0.95535x^{2}\\ & +0.0020008x^{3}+0.64328\exp\left(x\right), \end{array}$$

and this function has equivalent asymptotic properties. We therefore use this formula in place of log(1+erf(x)) in our learning code for x<−1. The torch code is given in Fig. 9, and can be used to replace any appearance of log(1+erf(x)) in code.

### Theoretical Limit.

In ref. 35, the authors measure the distribution of instability times for various orbital configurations. Taking an initial orbital configuration, the authors perturb the system by machine precision, and measure the instability time, and repeat. For each system, the authors then measure the mean instability time, μ (in log-space), as well as the SD, σ (in log-space, modeled as a log-normal). What this means is that we can define a “theoretical limit” to the accuracy with which we can predict the instability time, and this accuracy is bounded by σ, for we cannot predict the instability time for a given system better than within one σ SD on average. For the purposes of this paper, we simulate an optimal estimator by taking a particular instability time, and then making a random prediction for its instability time within one σ of the actual instability time. Ref. 35 found that σ, while it is different for different configurations, does not correlate to μ, so, for the numerical value of σ, we simply randomly select numerical σ values from those released for ref. 35. On average, these SDs are 0.43 dex.

## Data Availability

Simulation/numerical integration output data have been deposited in GitHub (https://github.com/MilesCranmer/bnn_chaos_model). Our inference model is publicly available in the SPOCK (https://github.com/dtamayo/spock) package. Data are currently publicly available on Zenodo (https://zenodo.org/record/5501473) (72). This work made use of several Python software packages: numpy (73), scipy (74), sklearn (75), jupyter (76), matplotlib (77), pandas (78), torch (71), pytorch-lightning (79), and tensorflow (80).

## Footnotes

*The mutual Hill radius $R_{H}$ is a relevant length scale within which the gravity of the planets dominates that of the star, $R_{H}\approx a_{1}{\left(\mu_{1}+\mu_{2}\right)}^{1/3}$, where $a_{1}$ is the inner planet’s semimajor axis, and $\mu_{1}$ and $\mu_{2}$ are each planet’s mass ratio relative to the star.

^‡^The hyperopt package is available at https://github.com/hyperopt/hyperopt.
